# Hypervirulent group A *Streptococcus* emergence in an acaspular background is associated with marked remodeling of the bacterial cell surface

**DOI:** 10.1371/journal.pone.0207897

**Published:** 2018-12-05

**Authors:** Jessica Galloway-Peña, Sruti DebRoy, Chelcy Brumlow, Xiqi Li, Truc T. Tran, Nicola Horstmann, Hui Yao, Ken Chen, Fang Wang, Bih-Fang Pan, David H. Hawke, Erika J. Thompson, Cesar A. Arias, Vance G. Fowler, Micah M. Bhatti, Awdhesh Kalia, Anthony R. Flores, Samuel A. Shelburne

**Affiliations:** 1 Department of Infectious Diseases Infection Control and Employee Health, The University of Texas MD Anderson Cancer Center, Houston, Texas, United States of America; 2 Department of Genomic Medicine, The University of Texas MD Anderson Cancer Center, Houston, Texas, United States of America; 3 Center for Antimicrobial Resistance and Microbial Genomics and Division of Infectious Diseases, UTHealth McGovern Medical School, Houston, Texas, United States of America; 4 Department of Bioinformatics and Computational Biology, The University of Texas MD Anderson Cancer Center, Houston, Texas, United States of America; 5 The Proteomics and Metabolomics Facility, The University of Texas MD Anderson Cancer Center, Houston, Texas, United States of America; 6 Department of Genetics, The University of Texas MD Anderson Cancer Center, Houston, Texas, United States of America; 7 Center for Infectious Diseases, UTHealth School of Public Health, Houston, Texas, United States of America; 8 Molecular Genetics and Antimicrobial Resistance Unit-International Center for Microbial Genomics, Universidad El Bosque, Bogota, Colombia; 9 Division of Infectious Diseases, Duke University Medical Center, Durham, North Carolina, United States of America; 10 Department of Laboratory Medicine, The University of Texas MD Anderson Cancer Center, Houston, Texas, United States of America; 11 Graduate Program in Diagnostic Genetics, School of Health Professions, The University of Texas MD Anderson Cancer Center, Houston, Texas, United States of America; 12 Department of Pediatrics, University of Texas Health Science Center McGovern Medical School, Houston, Texas, United States of America; New York Medical College, UNITED STATES

## Abstract

Inactivating mutations in the control of virulence two-component regulatory system (*covRS*) often account for the hypervirulent phenotype in severe, invasive group A streptococcal (GAS) infections. As CovR represses production of the anti-phagocytic hyaluronic acid capsule, high level capsule production is generally considered critical to the hypervirulent phenotype induced by CovRS inactivation. There have recently been large outbreaks of GAS strains lacking capsule, but there are currently no data on the virulence of *covRS*-mutated, acapsular strains *in vivo*. We investigated the impact of CovRS inactivation in acapsular serotype M4 strains using a wild-type (M4-SC-1) and a naturally-occurring CovS-inactivated strain (M4-LC-1) that contains an 11bp *covS* insertion. M4-LC-1 was significantly more virulent in a mouse bacteremia model but caused smaller lesions in a subcutaneous mouse model. Over 10% of the genome showed significantly different transcript levels in M4-LC-1 vs. M4-SC-1 strain. Notably, the Mga regulon and multiple cell surface protein-encoding genes were strongly upregulated–a finding not observed for CovS-inactivated, encapsulated M1 or M3 GAS strains. Consistent with the transcriptomic data, transmission electron microscopy revealed markedly altered cell surface morphology of M4-LC-1 compared to M4-SC-1. Insertional inactivation of *covS* in M4-SC-1 recapitulated the transcriptome and cell surface morphology. Analysis of the cell surface following CovS-inactivation revealed that the upregulated proteins were part of the Mga regulon. Inactivation of *mga* in M4-LC-1 reduced transcript levels of multiple cell surface proteins and reversed the cell surface alterations consistent with the effect of CovS inactivation on cell surface composition being mediated by Mga. CovRS-inactivating mutations were detected in 20% of current invasive serotype M4 strains in the United States. Thus, we discovered that hypervirulent M4 GAS strains with *covRS* mutations can arise in an acapsular background and that such hypervirulence is associated with profound alteration of the cell surface.

## Introduction

Invasive bacterial infections afflict >100,000 persons per year in the United States alone. The vast majority of these infections are caused by commensal microflora, which leads to the question of how asymptomatically carried bacteria also cause invasive disease [1–4]. One possible answer comes from the long standing observation that passaging of bacteria in mice can increase virulence, suggesting that interaction with the host immune system can augment infectivity [5–8]. Whole genome sequencing of isolates recovered from mouse challenge studies as well as bacteria causing human infection has shown that alterations in genes encoding regulatory proteins are a major mechanism by which bacteria achieve hypervirulence [9–14].

Group A *Streptococcus*, or GAS, causes millions of human infections per year worldwide [15]. Nearly 100 years ago, Rebecca Lancefield made the key observation that some, but not all, GAS strains increase in virulence after passage in mice and, once achieved, the hypervirulent phenotype persists [7]. The capacity of GAS strains to develop hypervirulence has been associated with severe and prevalent human GAS infections. For instance, the epidemic serotype M1 clone, called M1T1, which is often identified as the most common cause of GAS infection worldwide, readily converts to a hypervirulent phenotype in mice whereas the serotype M1 clone that M1T1 GAS replaced, does not [16, 17]. Studies over the past 15 years have established that GAS hypervirulence is mainly due to mutations in the control of virulence (*covRS*) two-component gene regulatory system [18–22]. The CovS sensor kinase, which primarily serves to phosphorylate its cognate response regulator CovR, is the most common site for such mutations [20, 21, 23]. Inasmuch as phosphorylated CovR represses a number of GAS virulence factors, such CovS mutations result in decreased CovR phosphorylation and thus increased GAS virulence factor production [23–27].

The vast majority of work on GAS hypervirulence has centered on M1T1 GAS in which the anti-phagocytic M protein and hyaluronic acid capsule have been shown to be necessary for emergence of hypervirulent GAS due to *covRS* mutations [28, 29]. However, recent surveys have identified a significant increase in acapsular GAS strains causing both mucosal and invasive infections, suggesting that GAS strains lacking hyaluronic acid capsule could develop hypervirulence mechanisms as well [30, 31]. We recently reported a cluster of invasive GAS infections due to acapsular serotype M4 strains [32]. Herein, we further study these strains to discover that CovS inactivation leading to a hypervirulent phenotype can occur in an acapsular background during human infection and results in vast transcriptional changes, including genes encoding key cell surface proteins whose transcript levels are not altered by CovS inactivation in serotype M1T1 GAS.

## Results

### Identification of a CovS-inactivated serotype M4 strain

**Fig 1:**
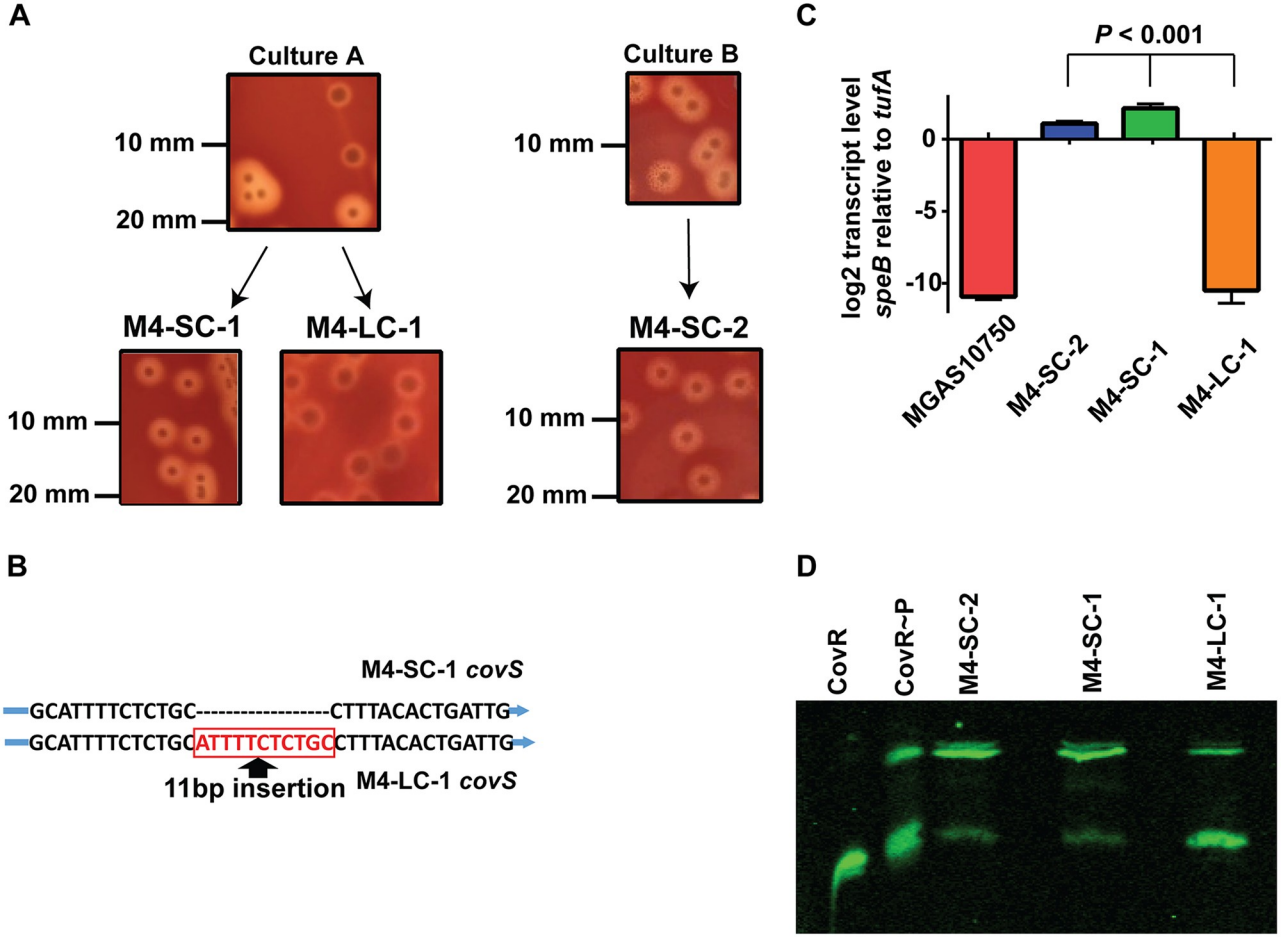
Description and characterization of M4 isolates used in this study. (A) Comparison of colony morphology for small colony (SC) and large colony (LC) isolates derived from patient cultures as described in the text. (B) Schematic of *covS* inactivation in strain M4-LC-1. (C) TaqMan qRT-PCR of *speB* at stationary phase for SC and LC variants along with the reference serotype M4 strain MGAS10750 which is known to not produce *speB* transcript due to a mutation in the regulator of proteinase B (*ropB*) gene [32]. Strains were grown in duplicate on two separate days and analyzed in duplicate for a total of 8 data points. Data shown are mean ± standard deviation. *P* value refers to analysis of variance. (D) Representative Phostag-Western blot (n = 2) using anti-CovR antibodies to measure CovR phosphorylation levels (CovR~P, upper band; CovR, lower band) in cell lysates of serotype M4 strains grown to mid-exponential phase. Recombinant/purified CovR/CovR~P was used as controls.

We reported on two patients who contracted invasive infection due to acapsular serotype M4 strains from a point-source outbreak previously described as isolates A and B [32]. However, upon working with the two cultures, we noted that one contained a mixture of large and small colony variants (culture A), whereas the other contained homogenous small colonies (culture B, Fig 1A, Table 1). Given that inactivation of either the *covR* or *covS* gene has been shown to generate a large colony phenotype in many GAS serotypes [19, 22, 33], we sequenced the *covRS* region of 15 separate large colony isolates and found that all contained an 11bp duplication (ATTTTCTCTGC) early in the *covS* gene (Fig 1B). This 11bp duplication results in a frame shift that inactivates CovS and has previously been identified in invasive GAS strains of various serotypes [20, 21, 34]. This finding is consistent with a mixed patient culture containing CovS-mutated bacteria arising from a CovS wild-type background, a clinical situation that we and others have previously reported [34, 35]. We stocked a small colony isolate from culture A to avoid artifact induced by serial laboratory passage and named this strain M4-SC-1 and did the same with a large colony strain which was named M4-LC-1 (Fig 1A). The small colony from culture B was named M4-SC-2. We performed deep sequencing (>1,000 fold) of strain M4-SC-1 and found no evidence of *covRS* mutations consistent with the concept of a pure CovRS+ culture. We confirmed the CovS-inactivated phenotype of strain M4-LC-1 by demonstrating markedly reduced transcript level of the broad spectrum streptococcal protease B (*speB*), a hallmark of CovS inactivation [17] (Fig 1C). Moreover, strain M4-LC-1 had significantly lower levels of phosphorylated CovR compared to strain M4-SC-1 and M4-SC-2 consistent with absence of CovS activity (Fig 1D) [23].

**Table 1:** Strains and plasmids used in this work.

Strain or plasmid	Description	Reference
**Strains**		
M4-SC-1	Invasive clinical isolate, serotype M4, CovRS wild-type	[32]
M4-SC-2	Invasive clinical isolate, serotype M4, CovRS wild-type	[32]
M4-LC-1	Invasive clinical isolate, serotype M4, co-isolated with M4-SC-1, CovS inactive	This study
M4-SC-1Δ*covS*	M4-SC-1 Δ*covS*::*spc*	This study
M4-SC-1Δ*mga*	M4-SC-1 Δ*mga*::*spc*	This study
M4-LC-1Δ*mga*	M4-LC-1 Δ*mga*::*spc*	This study
MGAS2221	Invasive clinical isolate, reference serotype M1, CovRS wild-type	[19]
2221Δ*covS*	MGAS2221 Δ*covS*::*spc*	[36]
MGAS10870	Invasive clinical isolate, reference serotype M3, CovRS wild-type	[9]
10870Δ*covS*	MGAS10870 Δ*covS*::*spc*	[23]
10750	Pharyngeal isolate, reference serotype M4, CovRS wild-type	[37]
10750Δ*covS*	MGAS10750 Δ*covS*::*spc*	This study

### Comparative whole genome analyses of the M4 strains

**Fig 2:**
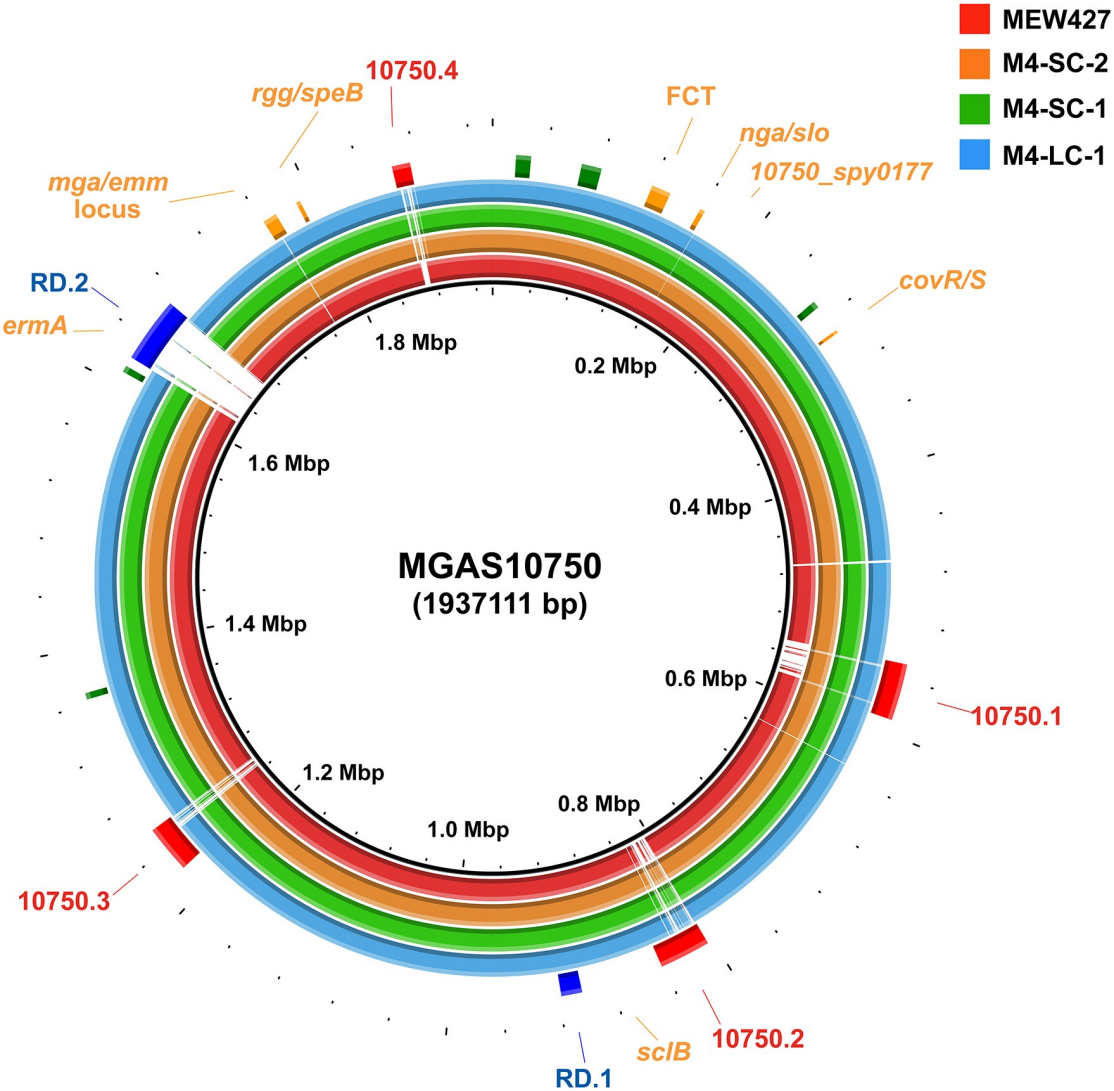
Comparative whole genome analysis of M4 Group A *Streptococcus* strains. Genomes of the three strains analyzed here (M4-SC-1, M4-SC-2, and M4-LC-1) along with the previously published M4 strain MEW427 [39] are shown in comparison to the serotype M4 strain MGAS10750 [37]. Genome scale in megabases (Mbp) is given in the innermost circle. Reference genome landmarks are displayed and include ribosomal operons (green), mobile genetic elements (red, 10750.1–4), and previously identified regions of difference (RD.1-2) [37]. Colored rings show BLASTN comparisons of strain MGAS10750 with strains as indicated in the figure legend.

Previous sequencing of M4-SC-1 and M4-SC-2 by a short-read approach had shown them to be genetically identical [32] when they were compared to the reference serotype M4 strain MGAS10750. However, this approach did not allow us to ascertain whether there were inter-strain differences in genetic regions absent in MGAS10750. Thus, we next used a combination of a short-read approach plus PacBio sequencing to complete the genomes of strains M4-SC-1, M4-SC-2, and M4-LC-1 (Fig 2). The only consistent difference between M4-LC-1 compared to the SC strains was the 11bp inactivating duplication in *covS*. Among the three strains, we also found variations in repeat regions in the *sclB* gene, which is known to be a hot-spot for GAS genetic variation [38], and in a hypothetical protein encoded by *10750_spy_0177* (S1 Table). Compared to strain MGAS10750, all three strains lacked the region of difference 2 (RD.2) which includes *ermA* encoding for erythromycin resistance (Fig 2). RD.2 is also absent in the other publically available, fully sequenced M4 strain MEW427 [39]. Consistent with the acapsular nature of M4 strains [40], the hyaluronic acid capsule-encoding genes *hasABC* were absent in all five strains. Thus, even when comparing complete genomes, the only consistent genetic difference separating the LC from the SC strains was the *covS* insertion.

### The CovS-inactive strain M4-LC-1 shows site specific increase in virulence

**Fig 3:**
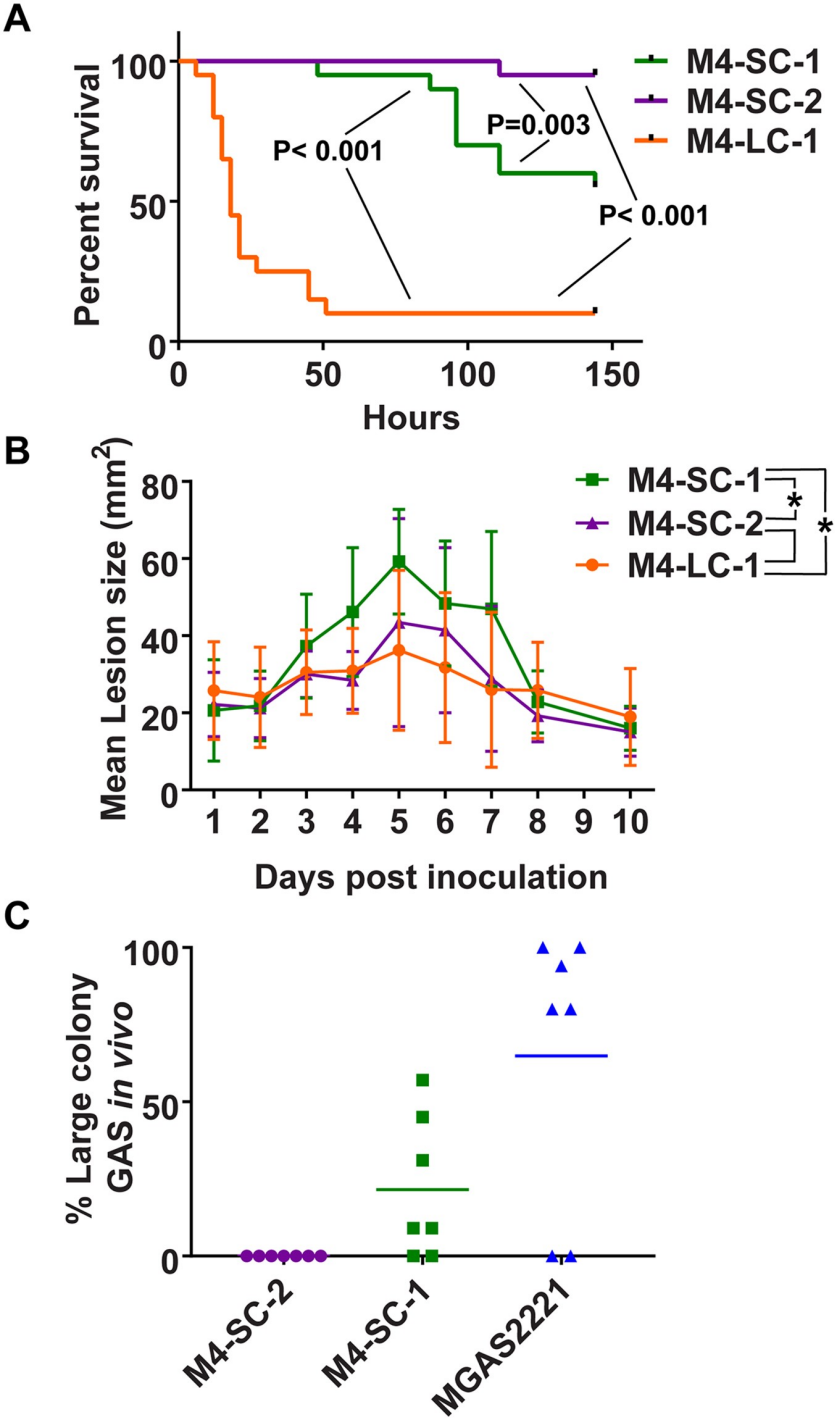
Effect of CovS inactivation on virulence of serotype M4 strains in murine models of infection. (A) 20 CD-1 swiss mice per strain were inoculated with 5 x 10^7^ colony forming units (CFUs) of the indicated GAS strains by intraperitoneal route and monitored to near-mortality. Kaplan Meier survival curves are shown with *P* values derived using a Mantel-Cox (log-rank) test and considered significant for a *P* value < 0.05 adjusted for multiple comparisons. (B). 20 SKH1-hr mice per strain were inoculated subcutaneously with 1 x 10^7^ CFUs of the indicated strains. Lesion size was measured daily, and groups were compared using two way ANOVA and considered significant for a *P* value < 0.05 adjusted for multiple comparisons. * indicates significant difference between strains connected by lines within figure legend (i.e. there was no statistically significant difference between strains M4-SC-2 and M4-LC-1). (C) In the subcutaneous model, 7 mice from each group were sacrificed at 72hrs, lesions harvested, and plated onto sheep blood agar. Plates were analyzed for the emergence of the large colony phenotype and reported as a percentage of the total CFUs recovered.

CovS inactivation increases the virulence of GAS in bacteremia models [18, 19, 36], but the impact of CovS inactivation on infection in an acapsular GAS strain has not been assessed. Thus, we challenged mice with the CovS wild-type strains M4-SC-1 and M4-SC-2 as well as the CovS-inactivated strain M4-LC-1 using an intraperitoneal (IP) bacteremia model. Consistent with CovS inactivation resulting in a hypervirulent phenotype, the survival times of mice challenged with M4-LC-1 were significantly shorter compared to M4-SC-1 and M4-SC-2 (*P*<0.001) (Fig 3A). There was also a statistically significant difference between strain M4-SC-1 and M4-SC-2 with the survival times being shorter in mice challenged with strain M4-SC-1 (P = 0.003).

Next, we challenged mice subcutaneously with the three strains as the impact of CovS inactivation in the skin/soft tissue infection model varies between serotypes [19, 41]. Contrary to what we observed for the bacteremia model, the CovS-inactivated strain M4-LC-1 created smaller lesions and was less virulent compared to strain M4-SC-1 (P<0.001, Fig 3B). Similar to the bacteremia challenge, M4-SC-1 caused significantly larger lesion sizes in the skin soft tissue model compared to strain M4-SC-2 (P<0.001; Fig 3B).

To determine whether hypervirulent, CovS-inactivated strains could emerge during infection, four days after inoculation we excised lesions from mice infected subcutaneously with strains M4-SC-1 and M4-SC-2, plated onto sheep blood agar plates, and assayed for colonies with altered morphology [29]. We also challenged mice with the serotype M1 strain MGAS2221 which is known to readily develop mutations in the CovRS system following mouse challenge as a positive control [19]. The total CFU per gram of lesion for strains M4-SC-1, M4-SC-2, and MGAS2221 ranged from 2.5x10^5 to 3.4X10^8 among all strains, however, there was no statistically significant difference in the total CFU count between strains (S1 Fig). The large colony phenotype was only detected in mice infected with strain M4-SC-1 (Fig 3C) and we could detect the CovS-inactivating 11bp insertion in the large colony isolates.

### CovS inactivation affects the Mga regulon and the FCT locus in strain M4-SC-1

**Fig 4:**
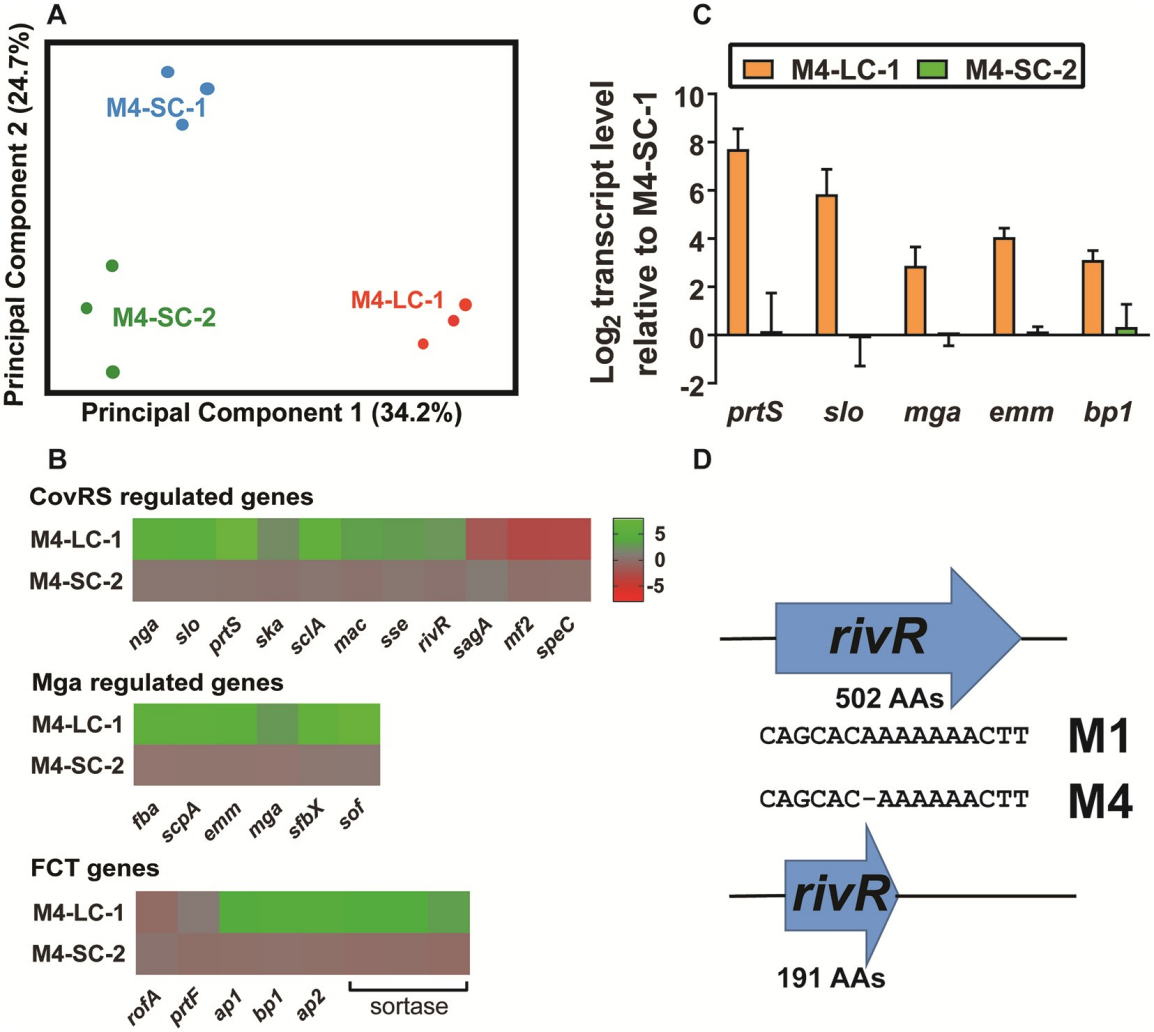
Characterization of the CovS transcriptome in serotype M4 GAS. (A) Principal component analysis showing distinct transcriptomes of strain M4-SC-1, M4-SC-2, and M4-LC-1, which are labeled. Each strain has three biological replicates. (B) Heat map of log_2_ transcript levels of select genes for the indicated strains relative to strain M4-SC-1. Genes are grouped as indicated. FCT = Fibronectin, collagen, T-antigen region. The color value scale is on the right. (C) Taqman qRT PCR analysis to verify the alteration in select gene transcript levels representing the typical and the atypical groups of genes affected by *covRS* mutation in the M4 strains. Data represents the mean ± standard deviation of strains grown in duplicate on two separate days and analyzed in duplicate (total of 8 data points). (D) Schematic representation of the single nucleotide change in serotype M4 strains that results in a truncated RivR protein compared to serotype M1.

To gain insight into the mechanism underlying our virulence observations, we next performed RNAseq analyses of strains M4-SC-1, M4-SC-2 and M4-LC-1 grown to mid-exponential phase in THY, in triplicate. Principal component analysis showed the transcriptomes of all three strains to be reproducible and distinct (Fig 4A). Differentially regulated genes were defined as those with a transcript level variance of ≥ 2-fold and a *P* value of <0.05 after Bonferroni correction for multiple comparisons. Consistent with the minimal genetic differences separating the two strains, only 21 genes had differential transcript levels when we compared strain M4-SC-1 to M4-SC-2 (6 genes increased; 15 genes decreased) (S2 Table). In contrast, we observed marked differences in the transcriptome of the CovS-inactivated M4-LC-1 strain when compared to both SC strains. Statistically significant differences were observed in transcript levels for 163 genes (114 genes increased; 49 decreased) and 172 genes (99 increased; 73 decreased) for strain M4-LC-1 compared to strains M4-SC-1 and M4-SC-2, respectively (S3 and S4 Tables).

In accordance with previous reports in other serotypes, the transcript levels of numerous genes known to be regulated by the CovRS system were increased in strain M4-LC-1 including those that encode PrtS, an IL-8 degrading enzyme, Ska, a plasminogen activating protein, and SLO, a pore forming toxin [18, 19, 33] (Fig 4B). However, in contrast to prior observations following CovS inactivation in other GAS serotypes, we observed marked increases in the transcript levels of genes encoding two groups of cell surface proteins [36, 42]. The first includes the critical anti-phagocytic cell surface M protein encoded by *emm*, the streptococcal C5a peptidase ScpA, the fibronectin-binding serum opacity factor Sof, and the fibrinogen-binding surface proteins SfbX and Fba. *Emm*, *scpA*, *sof*, *sfbX* and *fba* are part of the multi-gene activator (Mga) regulon [43, 44], and *mga* transcript was significantly increased in the CovS-inactivated strain compared to the CovS wild type strains (Fig 4B). Increased transcript levels of Mga-regulated genes and *mga* were confirmed using qRT-PCR (Fig 4C). Although CovRS has not previously been identified as influencing Mga activity, it is known that CovRS represses the transcript of the gene encoding the regulatory protein RivR [45] which has been hypothesized to increase Mga activity [46]. Of note, in our three M4 strains and in MGAS10750 and MEW427, *rivR* contains a single base pair deletion in a homopolymeric tract of six A nucleotides which results in an early stop codon at amino acid 192 of the RivR protein (functional RivR has 502 amino acids) (Fig 4D). A distinct, but similar, single base pair deletion in a homopolymeric tract of seven T nucleotides was recently identified in serotype M3 GAS strains and shown to result in RivR inactivation [47]. Thus, although *rivR* transcript levels were significantly higher in our CovS-inactivated strain compared to CovS wild-type strains (Fig 4B), RivR is most likely inactive in serotype M4 strains and thus not the cause of increased transcript levels of Mga regulon genes.

The second group of cell surface protein-encoding genes that showed elevated transcript levels in our CovS-inactivated strain but had not previously been identified as being part of the CovRS regulon are located in the FCT locus (Fibronectin and Collagen-binding proteins and T-antigen) (Fig 4B). These genes include *bp1*, *ap1*, and *ap2*, which encode the major and accessory pilus proteins, as well as sortases involved in pilus formation [48]. Gene content and regulation of the FCT region among GAS is highly varied, but pilus encoding genes have not previously been identified as being part of the CovRS or Mga transcriptome [36, 43]. M4 strains lack the pilus regulators MsmR and Nra, and the transcript level of *rofA*, which encodes a protein known to regulate the first gene in the FCT locus, was not significantly different between our CovS active and inactive strains (Fig 4B). Thus, the mechanism of elevation of the pilus encoding genes in strain M4-LC-1 is not clear. In summary, the transcriptome data indicate that CovS inactivation in our serotype M4 strain markedly alters the transcript levels of numerous key GAS cell surface protein encoding genes not previously identified as being part of the CovRS transcriptome.

### Identification of marked cell surface remodeling in strain M4-LC-1

**Fig 5:**
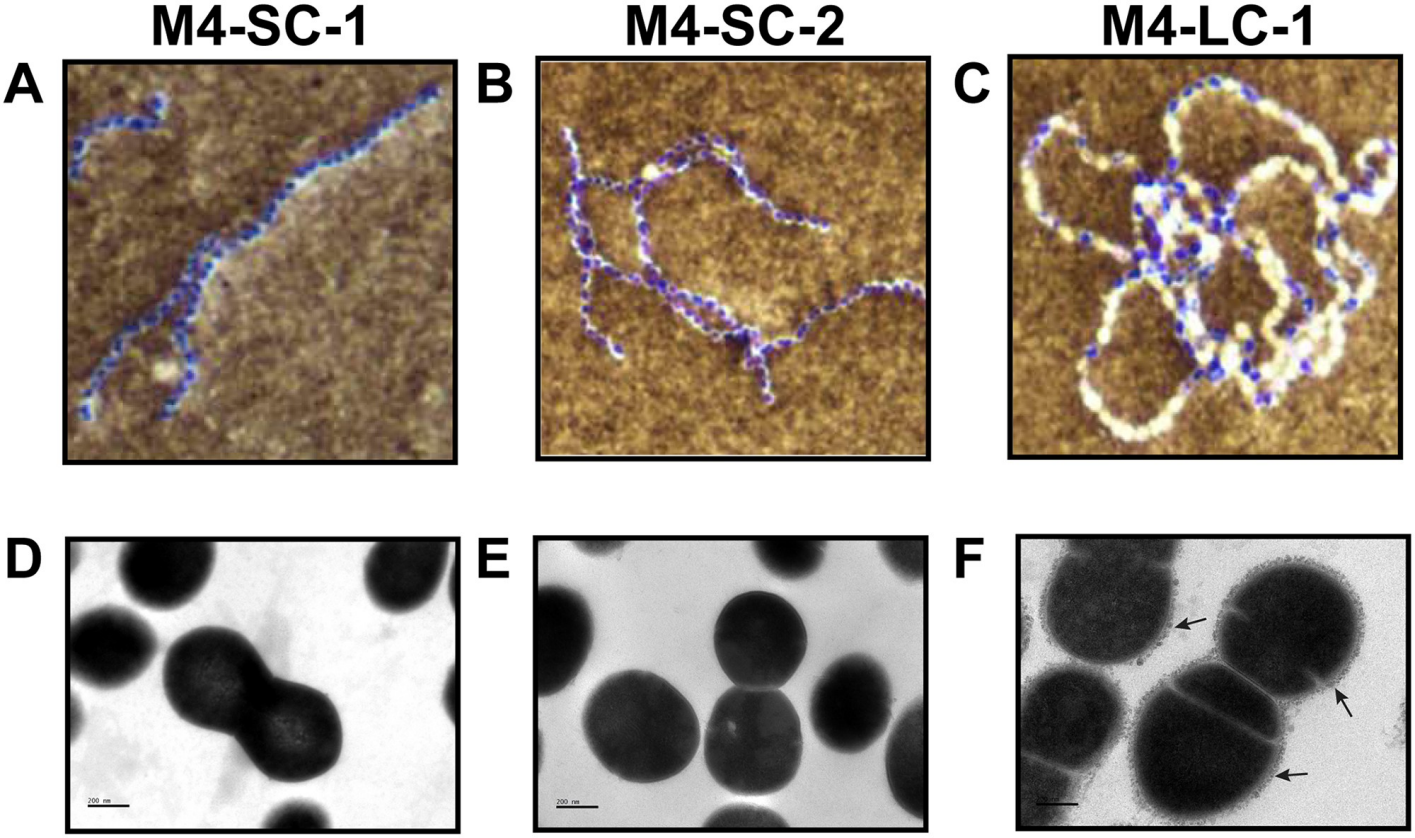
Strain M4-LC-1 has altered cell surface morphology. (A-C). Light microscopy of indicated strains following India ink and crystal violet staining. (D-F). Transmission electron micrographs of CovS intact strains (M4-SC-1 and M4-SC-2) show smooth cell surfaces, whereas CovS-inactivated strain M4-LC-1 has rough cell walls covered by protruding material (indicated by arrows). Sections in A-C are shown at a magnification of 100X and sections in D-F are shown at a magnification of 40,000 with bars indicated 200 nM.

We next sought to determine whether the transcript level differences observed in several genes encoding cell surface proteins in the CovS-inactivated strain structurally altered the GAS cell surface. To this end, we performed light microscopy and found that strain M4-LC-1 aggregated rather than form the long chains that are typical of GAS and were observed for strains M4-SC-1 and M4-SC-2 (Fig 5A–5C). We next performed transmission electron microscropy (TEM) to explore cell morphology and changes in cell envelope structure more closely. In strains M4-SC-1 and M4-SC-2, we observed cells with a smooth surface (Fig 5D and 5E). In contrast, M4-LC-1 displayed a rough cell surface with numerous projections (Fig 5F) not observed in the M4-SC-1 or M4-SC-2 strains. Thus, these data indicate that the transcript level differences associated with CovS inactivation is accompanied by marked alteration of the appearance of the GAS cell surface in an acapsular strain.

### CovS inactivation in serotype M4 GAS reproduces the M4-LC-1 phenotypes

**Fig 6:**
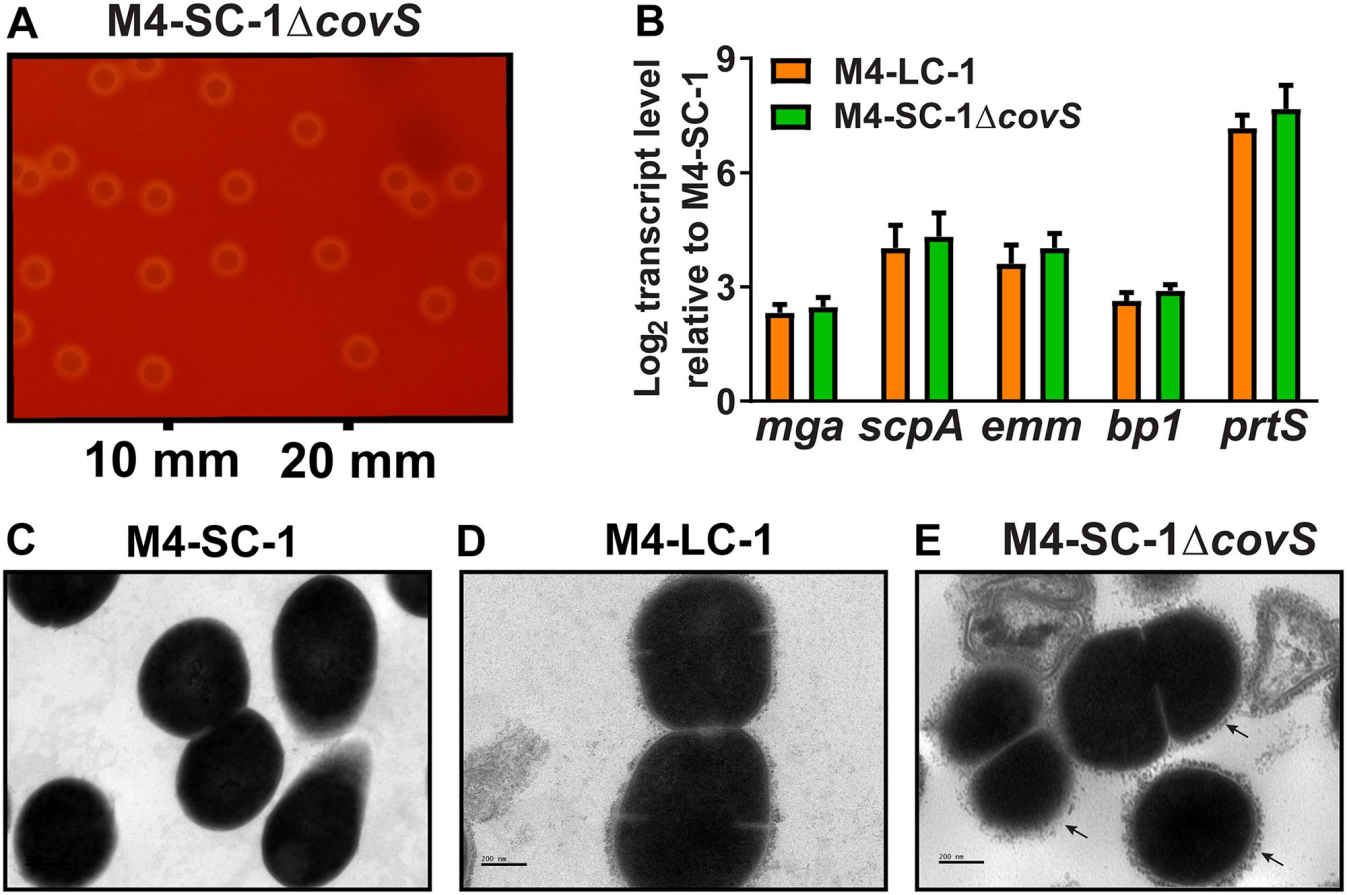
Targeted CovS inactivation recapitulates the M4-LC-1 phenotype. (A) Targeted inactivation of *covS* in strain M4-SC-1 produced a large colony phenotype analogous to that seen in strain M4-LC-1 (see Fig 1A for comparison). (B) Select gene transcript levels in the naturally occurring (M4-LC-1) and isogenic (M4-SC-1Δ*covS*) CovS mutants compared to the wild type CovS intact strain M4-SC-1. Data graphed are mean ± standard deviation of two biological replicates, with two technical replicates, done on two separate days (n = 8). (C-E) Transmission electronic microscopy at 40,000x magnification of indicated strains showing that targeted inactivation of *covS* reproduces the rough surface phenotype (indicated by arrows) seen in strain M4-LC-1.

Although our whole genome data suggested that the main difference between strain M4-LC-1 and M4-SC-1 was the 11bp duplication in *covS*, it remained possible that the differences observed between the two strains were not mediated or only partly mediated by CovS. Thus, to directly test the contribution of CovS to the M4-LC-1 phenotypes, we used targeted mutagenesis to create strain M4-SC-1Δ*covS*. M4-SC-1Δ*covS* demonstrated the same large colony morphology with a small zone of hemolysis on blood agar as seen in M4-LC-1 (Fig 6A). Moreover, qRT-PCR analyses validated upregulation of *emm*, *mga*, *scpA*, *bp1* and *prtS* in M4-SC-1Δ*covS* compared to the wild-type CovS intact strain M4-SC-1, comparable to that seen in M4-LC-1 (Fig 6B). Finally, by TEM, M4-SC-1Δ*covS* also displayed the same rough cell envelope features observed in strain M4-LC-1 (Fig 6C–6E). Thus, we conclude that the characteristics separating M4-LC-1 from M4-SC-1 are due to CovS inactivation.

### CovS inactivation increases the abundance of Mga-regulated cell surface proteins

**Fig 7:**
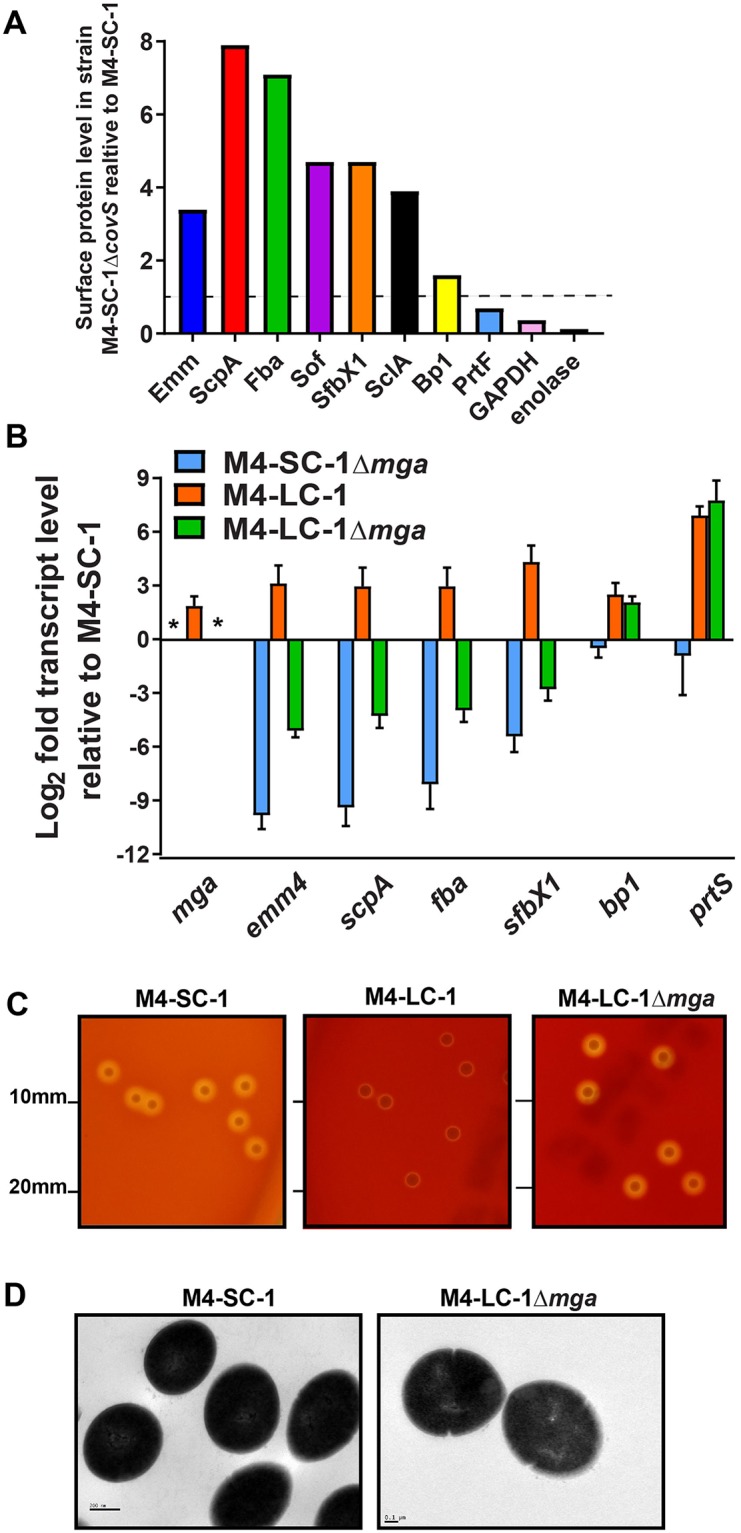
Mga is required for the M4-LC-1 surface phenotype. (A) Relative abundance of cell-surface proteins in isogenic M4-SC-1Δ*covS* mutant compared to the wild type CovS intact M4-SC-1 strain as determined by MassSpec analysis of shaved surface proteins. (B) Select gene transcript levels in the naturally occurring CovS mutant (M4-LC-1) and the isogenic *mga* mutants in the wild type CovS strain M4-SC-1 (M4-SC-1Δ*mga*) and the CovS mutant strain (M4-LC-1Δ*mga*), compared to the wild type CovS intact strain M4-SC-1. Data graphed are mean ± standard deviation of two biological replicates, with two technical replicates, done on two separate days (n = 8). * = no transcript level detected for *mga* in the two Δmga strains. (C) Targeted inactivation of *mga* in strain M4-LC-1 reverts the large colony phenotype to that seen for strain M4-SC-1. (D) Transmission electronic microscopy at 40,000x magnification of the isogenic *mga* mutant in the naturally occurring CovS mutant strain (M4-LC-1Δ*mga*) showing that targeted inactivation of *mga* causes reversion of the cell surface phenotype to that seen for the wild type CovS intact strain M4-SC-1.

To identify the basis of the altered cell surface morphology observed following CovS inactivation, we compared the proteins present on the surface of M4-SC-1Δ*covS* to that of M4-SC-1 using mass spectrometry. Exponentially growing strains were harvested and treated with trypsin in order to shave the surface proteins [49]. Peptides released were identified by LC-MS/MS. The CovS-inactive mutant, M4-SC-1Δ*covS*, showed increased surface presence of multiple proteins that are part of the Mga core regulon such as M protein, ScpA, and Sof (Fig 7A) [43]. In addition, there was also increased amounts of SclA, a collagen binding protein [50] which has been shown to be regulated by Mga [51]. In terms of the FCT locus, which is not known to be regulated by Mga, we detected more of the pilus subunit protein Bp1 in the CovS-inactive mutant, which was in concert with the increased transcript levels of *bp1* observed upon CovS inactivation (Fig 4B). In contrast, we did not observe changes in PrtF (Fig 7A), a surface-localized fibronectin-binding protein [52], transcript levels of which was unchanged in our RNAseq analysis (Fig 4B). The surface glycolytic enzymes enolase and GAPDH [53, 54] showed no relative difference in abundance between the strains.

To test the hypothesis that Mga contributes to the increased transcript levels of genes encoding multiple cell surface proteins following CovS inactivation, we then generated isogenic *mga* mutants in the M4-SC-1 and M4-LC-1 backgrounds. Consistent with our hypothesis, Mga inactivation in the CovS-inactive strain M4-LC-1 markedly decreased transcript levels of *emm4*, *scpA*, *fba*, *sof*, and *sfbX1* (compare orange and green bars in Fig 7B). However, the transcript levels of these genes was higher in strain M4-LC-1Δ*mga* compared to M4-SC-1Δ*mga* (compare blue and green bars in Fig 7B) indicating that even in the absence of Mga, CovS inactivation increases transcript levels of the aforementioned genes. Inactivation of Mga did not alter the transcript levels of *bp1* which encodes a pilus subunit and is part of the FCT locus, but whose transcript level was increased by CovS inactivation (Fig 7B). Inactivation of Mga in the M4-LC-1 strain resulted in reversion of the large colony morphology to that similar to the CovS wildtype strain M4-SC-1 (Fig 7C). Moreover, electron micrographs showed the surface of M4-LC-1Δ*mga* to be smooth (Fig 7D) like that of M4-SC-1. Thus, we conclude that a significant proportion of the alterations observed on the cell surface upon CovS inactivation in M4-SC-1 is mediated via Mga.

### Upregulation of the Mga regulon and pilus genes following CovS inactivation is observed in serotype M4 but not M1 or M3 strains

**Fig 8:**
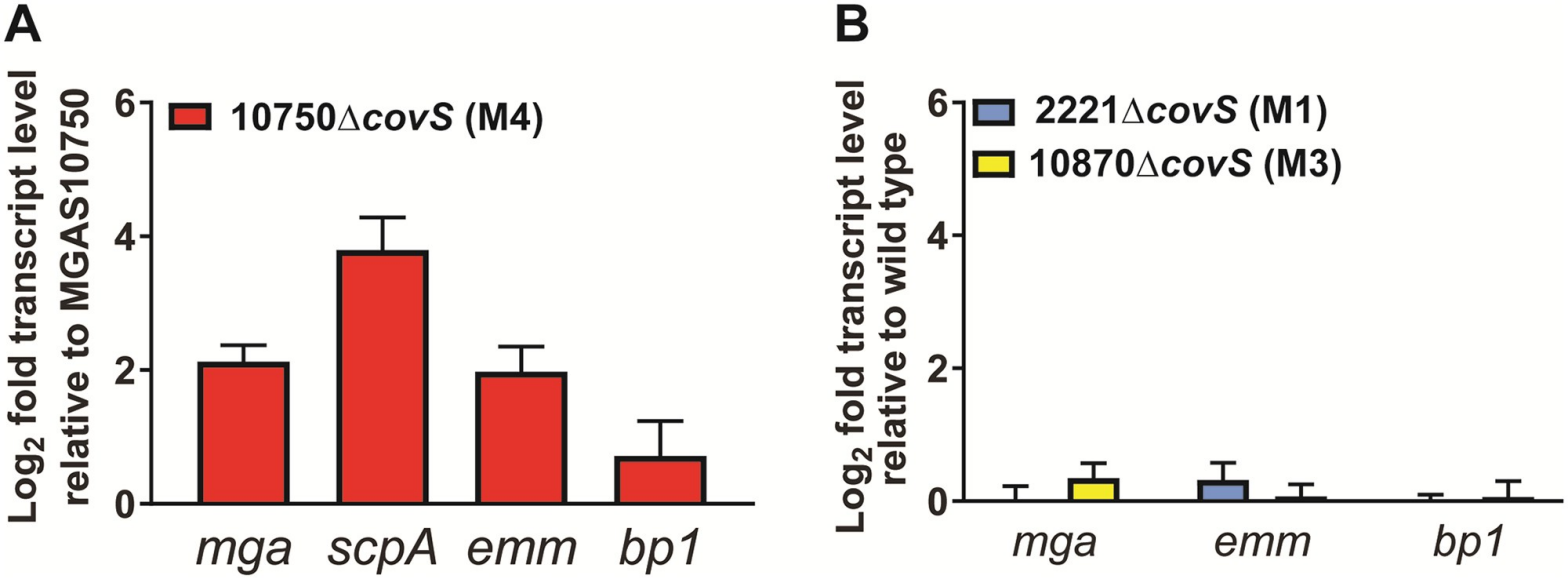
Activation of Mga regulon and pilus genes are not observed in M1 or M3 GAS strains. Significant upregulation of transcript levels of *mga*, *scpA*, *emm* and *bp1* is observed in isogenic *covS* mutants of serotype M4 strain MGAS10750 (A), but not for serotype M1 strain MGAS2221 or serotype M3 strain MGAS10870 (B). All data points represent two biological replicates, with two technical replicates, done on two separate days (n = 8).

To determine whether our finding of increased transcript levels of *mga*, the genes in the Mga-regulon, and the FCT region following CovS inactivation was limited to strain M4-SC-1, we inactivated CovS in strain MGAS10750 which is considered the reference serotype M4 strain [37]. Similar to what we observed for strain M4-SC-1, deletion of CovS in strain MGAS10750 resulted in increased transcript levels of *mga*, the Mga-regulated genes *scpA* and *emm* and the pilus gene *bp1* (Fig 8A). Previous transcriptome analyses of M1 and M3 strains did not report significant differences in transcript levels of *mga* or Mga-regulated genes or the FCT region genes following CovS inactivation [18, 19, 23, 36, 55]. To reconfirm these findings, we used qRT-PCR to test *mga*, *emm* and *bp1* levels in wild-type and CovS-inactivated serotype M1 and M3 strains. Consistent with previous reports and in contrast to the M4 strains, we observed no significant differences in *emm*, *mga* or *bp1* levels for the M1 or M3 CovS-inactivated strains compared to wild-type (Fig 8B). These data indicate that the profound effects on the Mga regulon and the FCT region observed following CovS inactivation in serotype M4 GAS is not characteristic of the encapsulated GAS serotypes in which CovS inactivation has been studied.

### CovRS mutation is common in invasive serotype M4 strains

Recently, short-read, whole genome sequencing data were published for nearly 1,500 GAS isolates causing invasive infection in the United States in 2015 [56]. We analyzed the data for the 54 serotype M4 isolates from this collection, all of which lack the *hasABC* operon, and identified 11 isolates (~20%) with mutations known or predicted to affect CovRS function (S5 Table). The mutations include amino acid substitutions in CovR important for CovR function, deletions in *covS* similar to that observed in strain M4-LC-1, and amino acid substitutions in CovS known to decrease CovS auto-phosphorylation. These findings are in concert with a previous study of 17 invasive, acapsular M4 strains from Australia which identified CovRS mutations in eight isolates [31]. Taken together, these data indicate that mutations in the *covRS* system as we observed in our mini-outbreak strain occur commonly among unencapsulated serotype M4 isolates causing invasive disease in humans.

## Discussion

Adaptation to overcome the challenges posed by the host during infection is a major aspect of bacterial pathogenesis and essential for the continued success of numerous important bacterial pathogens. Although Dr. Lancefield described that passage of GAS in mice resulted in hypervirulence some 100 years ago [7], it was not until the early 2000s that inactivating mutations of the CovRS system were identified as the major cause driving the emergence of the GAS hypervirulent phenotype [22]. Currently it is thought that upregulation of capsule engendered by CovRS inactivation is a key contributor to the ability of GAS to develop hypervirulence [28, 29]. However, recent increases in invasive and non-invasive infections by acapsular GAS [30, 31, 57, 58] suggest that hypervirulence might be achieved even in the absence of capsule. A recent study demonstrating the mutually exclusive interaction between GAS capsule and HylA expression in 17 invasive M4 GAS isolates in Australia, found more than 50% of the M4 isolates harbored mutations within *covRS* and were *speB* deficient [31]. Additionally, Turner et al. suggested that acapsular M89 strains with increased NADase and SLO activity could potentially be related to mutations in *covS* or *rocA* found in a small percentage of the isolates, however, they did not go on to validate this hypothesis [59]. Furthermore, they reported no difference in human blood survival between emergent M89 strains and non-emergent M89 strains, whether they included or excluded strains with *covR/S* or *rocA* mutations. So, although mutations in CovRS have been reported in acapsular M4 and M89 strains previously, the impact of CovRS mutations on virulence or global gene expression has not been directly assessed using isogenic mutant strains. Herein, we demonstrate that CovRS inactivation in acapsular GAS strains can lead to the emergence of hypervirulence thereby extending our understanding of a key aspect of GAS pathogenesis.

In addition to demonstrating that GAS hypervirulence can occur due to CovS inactivation in an acapsular background, we also found that inactivation of CovS in serotype M4 strains resulted in marked increases in transcripts of several cell surface protein-encoding genes including members of the Mga regulon and the FCT locus. Importantly, this phenomenon has not been observed following CovS inactivation in encapsulated serotype M1 and M3 strains [19, 36, 55]. The significantly elevated transcript levels of *mga* and Mga regulon genes such as *emm* following CovS inactivation in serotype M4 strains indicate a heretofore unappreciated link between the CovRS and the Mga regulons in this serotype.

The transcript levels of Mga-regulated genes in the *covS mga* double mutant were lower compared to those observed in the *covS* mutant suggesting that derepession of Mga following CovS inactivation contributes to the high transcript levels of the Mga-regulon genes observed in strain M4-LC-1. In an M1 strain, it has been proposed that the CovRS-regulated protein RivR or its associated small RNA *rivX*, could mediate crosstalk between CovR and Mga [46]. However, targeted RivR mutagenesis in the serotype M1 background did not impact *mga* or *emm*, and RivR is naturally inactive in M3, and likely M4 strains, and thus appears unlikely to link the two systems in most GAS strains [45, 60]. Thus, further study will be needed to determine how the CovRS and Mga regulons interact in M4 strains and whether this interaction is also seen in other unencapsulated GAS serotypes.

The mechanism linking CovS to the FCT region in our M4 strains is also unknown given that the FCT region genes are not traditionally considered part of either the CovRS or Mga regulons [43, 44, 55]. Our data also shows that a *covS mga* double mutant and a *covS* single mutant have similar transcript levels of *bp1*, which encodes a pilus subunit and is part of the FCT locus, indicating that Mga derepression does not mediate the increase in FCT locus transcript levels observed following CovS inactivation. Regulation of FCT region genes is complex and likely differs between serotypes [61, 62]. The best studied proteins influencing transcription of FCT genes belong to the RofA and RofA-like protein (RALP) family with RofA upregulating transcription of several FCT region genes [63]. We observed no difference in *rofA* transcript level following CovS inactivation and other RALP family proteins such as RivR and Ralp3 appear to have inactivating mutations in our serotype M4 strains.

By TEM, the cell surface of M4-LC-1, but not its parental strain, displayed protruding hair-like structures. Previous TEMs of GAS have suggested that such structures are composed of M protein which would be consistent with the marked upregulation of *emm* observed in strain M4-LC-1 [64]. A study of a serotype M22 strain also observed that deletion of *mga*, but not *emm*, resulted in a smooth surface and suggested that other Mga-regulated cell surface proteins also contribute to GAS cell surface morphology [65]. We identified greater amounts of multiple Mga-regulated proteins on the surface of the isogenic CovS mutant compared to the CovS-intact wild type M4-SC-1. Moreover, a *covS mga* double mutant (M4-LC-1Δ*mga*) displayed a smooth cell surface similar to that of M4-SC-1. Therefore, it seems that the material observed on the GAS cell surface in the CovS-inactivated M4-LC-1 consists mostly of Mga-regulated cell surface proteins. This combination of unique regulatory changes and marked reorganization of the GAS cell surface following CovS inactivation in strain M4-SC-1 may be envisioned as a means to achieve hypervirulence in the absence of capsule.

In the bacteremia model, M4-LC-1 was markedly more virulent than both CovS wild-type strains which is fully consistent with studies of CovS inactivation in other GAS serotypes following IP inoculation [18–20]. The higher rate of near-mortality observed in mice infected with strain M4-LC-1 despite the absence of capsule is likely due to the large number of virulence factor encoding genes whose transcript levels were increased including *emm*, *prtS*, *scpA*, and *slo*. Additionally, the upregulation of genes from the FCT locus presumably would result in increased pilus production, but pilus production can either increase or decrease GAS virulence depending on strain and infection site [66–68]. It is unclear, however, how CovS inactivation affects dissemination in M4 strains as blood and organs were not collected for bacterial cell counts following IP inoculation.

Interestingly, mice infected with strain M4-SC-1 had higher mortality compared to strain M4-SC-2 in the IP model with the differences becoming apparent some 4 days after inoculation consistent with emergence of a hypervirulent variant following infection. Indeed, we observed similar increased virulence of strain M4-SC-1 relative to M4-SC-2 following subcutaneous inoculation and were selectively able to recover CovS-mutated strains from mice infected with M4-SC-1. DiRita et al. showed that a combination of CovR-intact and CovR-inactivated strains were synergistic in producing larger lesions sizes [22]. We hypothesize that during infection, a CovS-inactive form of M4-SC-1 emerges to produce a combination of CovS-wild type and CovS-mutated strains which together produce the larger skin lesions relative to both M4-SC-2 and M4-LC-1. Such a hypothesis is supported by the observation that the lesion sizes did not begin to diverge until day 3 of the experiment (Fig 3B). However, as histopathology studies were not performed during the subcutaneous model, we cannot distinguish if differences between lesion sizes are due to inflammatory cell recruitment. Given that strain M4-SC-1 and M4-SC-2 appeared genetically identical and were isolated from distinct patients following a point source transmission, these data suggest that perhaps a yet to be detected genetic or non-genetic mechanism differentiates the two strains and the ability to develop hypervirulence via CovS inactivation. While multiple virulence factors are likely to play distinct roles in invasive versus mucosal infection caused by M4 strains, it is unknown what the key factors are for each type of infection. Therefore, the specific mechanisms of virulence in different infection models are an important area of future study.

In summary, we have demonstrated that hypervirulence enabled by CovS inactivation can occur in GAS strains lacking hyaluronic acid capsule and has occurred in 20% of recently sequenced, invasive serotype M4 isolates in the United States [56]. In M1 and M3 strains, a key result of CovS inactivation is thickening of the capsule. In acapsular M4 strains, the strong upregulation of Mga-dependent cell surface protein-encoding genes following CovS inactivation can be envisioned as a parallel mechanism to achieve hypervirulence stemming from a common genetic event. Given that capsule-negative strains are observed in a variety of medically important bacteria [69–71], our finding of novel rewiring of genetic circuits in acapsular GAS may have heretofore unappreciated parallels in numerous pathogens.

## Materials and methods

### Ethics statement

This study was carried out in accordance with the recommendations in the Guide for the Care and Use of Laboratory Animals of the National Institutes of Health. The protocol was approved by MD Anderson Animal Care and Use Committee (Protocol Number: 00001550-RN00). All efforts were made to minimize suffering.

### Bacterial strains, growth conditions, and mutant generation

Bacterial strains used in this study are shown in Table 1. Strains were grown in a nutrient-rich standard laboratory medium (Todd-Hewitt broth with 0.2% yeast extract (THY)) at 37 °C with 5% CO_2_. Phenotypic characterization was performed following growth on sheep blood agar (BSA) plates. Non-polar insertional mutagenesis with a spectinomycin resistance cassette was employed to obtain isogenic *covS* mutants in the M4-SC-1 and MGAS10750 strains, and isogenic *mga* mutants in the M4-SC-1 and M4-LC-1 strains respectively, as previously described for MGAS10870 [42]. Analysis of CovR phosphorylation levels was performed using Phos-Tag gels as previously described [72].

### Genome sequencing

Genomic DNA (gDNA) was extracted from a single colony grown overnight in THY. High-quality gDNA was isolated using the MasterPure Kit (Illumina). Complete whole genomes were assembled using a combination of long-read PacBio data and short-read Illumina sequencing (250bp paired end) as previously described [10]. Verification of all genomic differences identified between various strains was performed using Sanger based sequencing. Deep sequencing of strain M4-SC-1 was performed using the TruSeq DNA PCR-Free Library Prep Kits followed by MiSeq, without performing whole genome amplification, which could introduce bias to DNA representation. A minimal sequencing depth of 1,000 fold was obtained across the genome, which ensured sensitive detection of any sequence variation of greater than 0.2% population frequency.

### Transcript level analysis

RNA was purified from various GAS strains grown to indicated growth phase using an RNeasy mini kit (Qiagen). 1 μg of RNA per sample was converted to cDNA using a High Capacity Reverse Transcription Kit (Applied Biosystems). TaqMan real-time qRT-PCR (primers and probes are listed in S6 Table) was performed on an Applied Biosystems Step-One Plus System as described [72]. All samples were done at least in duplicate on two separate occasions and analyzed in duplicate.

For RNASeq analysis, strains were grown in triplicate to mid-exponential phase in THY and RNA was isolated as for TaqMan qRT-PCR. RNAseq data analysis was performed as described using the M4-SC-1 genome as template [72]. 79 of 1853 (4.3%) genes were excluded from the analysis because of low-expression levels. Transcript levels were considered significantly different if the mean transcript level difference was ≥ 2.0-fold and the final, adjusted *P* value was less than 0.05 using Bonferroni correction.

### Animal experiments

For the bacteremia model, 20 female outbred CD-1 Swiss mice per strain (Harlan-Sprague-Dawley) were injected intraperitoneally with 5.0 x 10^7^ GAS colony forming units (CFU) per inoculum and monitored for near-mortality humane endpoints. For the first 72 hours post-inoculation, mice were monitored every 3 hours for signs of near-mortality such as lethargy, weight loss, anorexia, and hunching. After the first 72 hours, the mice were then monitored every 12 hours for the duration of the experiment. Mice were euthanized directly upon observation of moribund symptoms, however, approximately 15% of mice were found dead from the infection before an observation of near-mortality symptoms meeting the criteria for euthanasia was made. At the discretion the veterinary staff, a single dose of sustained release buprenorphine was given some of the mice to minimize pain during the first 24–72 hours of the experiment. The remaining mice were sacrificed humanely at day 7. Differences in survival were calculated using a Mantel-Cox (log rank) analysis with a *P* value of < 0.05 considered statistically significant.

For the skin/soft tissue infection model, immunocompetent hairless SKH1-hr female mice (Charles River BRF) were employed because the lack of hair facilitates lesion monitoring and excision. Twenty mice per group were inoculated subcutaneously with 1x10^7^ CFU per inoculum, and measured every 24 hours for lesion size as well as monitored for moribund symptoms. Although the GAS dose and lesion site were not expected to cause near-mortality or interfere with basic activities, approximately 3% of mice were euthanized upon presentation of near-mortality symptoms (same as described for bacteremia). Buprenorphine was given to mice at the discretion of the veterinary staff to prevent and/or relieve pain. The remaining mice were sacrificed humanely at day 10. Groups were compared using a two way ANOVA and considered statistically significant for a *P* value < 0.05. Emergence of hypervirulent strains was performed as described [29] using the subcutaneous challenge model followed by euthanasia and lesion excision after 72 hours for 5 mice per strain. GAS colonies were recovered from the lesion by sample homogenization in PBS and subsequent plating onto BSA. The *covRS* operon was amplified from single colonies via PCR and subjected to Sanger sequencing.

### Microscopy experiments

For light microscopy, a drop of India ink was applied to a microscope slide and an inoculating loop was used to transfer organism from the agar plate to the slide and mix it into the drop of India ink. The slide was air dried and then saturated with a Gram’s crystal violet for 1 minute and rinsed gently with distilled water and allowed to air dry. Light microscopy was performed using an Olympus BX45 scope with a 100X Plan N oil immersion objective. The images were captured using an Olympus DP27 camera and Olympus cellSense Standard Software (v1.15). To visualize the whole cell morphology of bacterial cells using transmission electron microscopy, streptococcal cells were grown in THY broth at 37°C for 12 hrs, collected by centrifugation, washed in Millonig’s buffer (pH 7.0) and subsequently fixed with 2% glutaraldehyde. The cells were postfixed in a suspension of 1% osmium tetroxide and Millonig’s buffer. After dehydration through an ascending series of ethanol solutions (50%, 70%, and 100%), the cells were soaked in propylene oxide. The cells were then infiltrated with LX-112 resin, imbedded in BEEM capsules, and polymerized overnight at 70 °C. Sections (500 nm) were prepared using a Leica Ultracut R microtome and stained with 0.5% Toluidine Blue. Thick and thin sections (120 nm and 80 nm, respectively) were done using a DiATOME diamond knife, floated on mesh copper grids, stained in Reynold’s lead citrate, rinsed and dry at 70 °C. TEM images were obtained using Jeol 1200 transmission electron microscope with a Gatan digital camera.

### Bacterial surface analysis

For analysis of cell surface proteins, bacteria were plated overnight onto blood agar and colonies were grown at 37°C in 200 ml of THY in the presence of 5% CO_2_ until an OD_600_ of 0.5. Bacteria were harvested by centrifugation at 3500 × *g* for 10 min at 4 °C and washed twice with PBS. Cells were suspended in 800 μl of PBS containing 40% sucrose (pH 7.4). Digestions were carried out with 10 μg trypsin (Promega, Madison, WI) for 30 min at 37°C. Validation that the cells maintained their cellular integrity during treatment was determined by plating bacterial strains before and after trypsin digestion to assure their CFUs were unchanged. Bacterial cells were then spun down at 3,500*g* for 10 min at 4°C and then spun at 14000Xg for 10min to remove any cell debris. Proteins were acetone precipitated (5:1) overnight at -20C and digested with 200–500 ng modified trypsin (sequencing grade, Promega, Madison WI) in the presence of RapiGest (Waters, Milford MA) for 18 hrs at 37°C. Resulting peptides were analyzed by high-sensitivity LC-MS/MS on an Orbitrap-Fusion mass spectrometer (Thermo Scientific, Waltham MA). Proteins were identified by database searching of the M4-SC-1 genome using Mascot (v 2.6.2, Matrix Science, London, UK). Typical search settings are: mass tolerances, 10 ppm precursor, 0.8d fragments; variable modifications, methionine sulfoxide, pyro-glutamate formation; up to 2 missed cleavages. FDR estimates were from Proteome Discoverer (v 1.4, Thermo Scientific), peak counting comparison was performed with Scaffold (Proteome Software, Portland OR).

### Analysis of invasive serotype M4 strains for *covRS* mutations

Sequencing data for 54 invasive serotype M4 strains recently sequenced by the Centers for Disease Control and Prevention [56] was accessed from BioProject PRJNA395240 SRA archives. The reads were subsequently mapped to *covRS* sequence of reference genome MGAS10750 with bowtie2 v2.2.3 with ‘sensitive’ preset parameters. The read alignment files were subject to duplicate reads removal using PICRAD v2.9.0–1 (Broad Institute, Cambridge, MA) Variant calling using Samtools v1.4 and SNP filtering (allele depth greater than 10 and genotype call quality score greater than 10) using bcftools v1.6 [73, 74]. The *covRS* consensus sequences of each strain generated based on mapping were then aligned with MUSCLE algorithm integrated in Geneious (Biomatters Ltd, NJ, USA) with default parameters.

## Supporting information

S1 Table Genetic differences among the clinical M4 strains in this study as determined by whole genome sequencing and confirmed via Sanger sequencing. (XLSX)

S2 Table Genes differentially expressed between M4-SC-1 and M4-SC-2 by RNA-seq analysis. (XLSX)

S3 Table Genes differentially expressed between M4-SC-1 and M4-LC-1 by RNA-seq analysis. (XLSX)

S4 Table Genes differentially expressed between M4-SC-2 and M4-LC-1 by RNA-seq analysis. (XLSX)

S5 Table CovRS mutations in other publically available M4 strains. (XLSX)

S6 Table Primers and probes used in this study. (XLSX)

S1 Fig Total bacterial counts from excised lesions after subcutaneous infection. (TIF)
