# Supplementary figures and images for: Hypervirulent group A Streptococcus emergence in an acaspular background is associated with marked remodeling of the bacterial cell surface
Source: PLoS One. 2018 Dec 5;13(12):e0207897. doi: 10.1371/journal.pone.0207897 (PMC6281247; doi:10.1371/journal.pone.0207897)

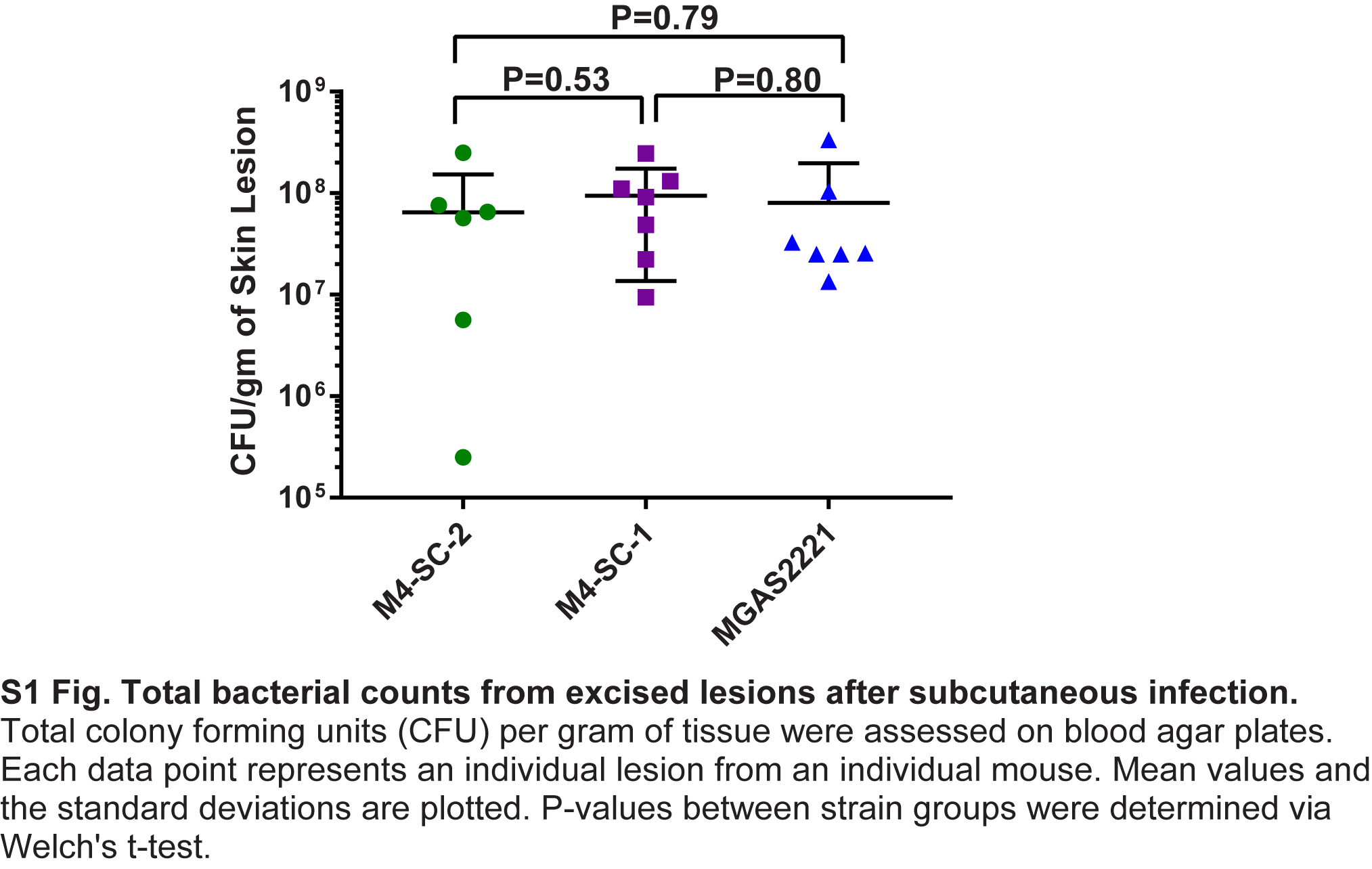

Supplement: S1 Fig — (TIF) [file pone.0207897.s007.tif]
